# Stroke, Fever, and Clot Microbiology Analysis: A Case Report

**DOI:** 10.7759/cureus.84782

**Published:** 2025-05-25

**Authors:** Mariana Baptista, Pedro Tavares de Almeida, Gabriela Abreu, Guilherme Jesus, Tiago Gregório

**Affiliations:** 1 Internal Medicine Department, Unidade Local de Saúde de Gaia e Espinho, Vila Nova de Gaia, PRT; 2 Neurology Department, Unidade Local de Saúde de Gaia e Espinho, Vila Nova de Gaia, PRT; 3 Microbiology Department, Unidade Local de Saúde de Gaia e Espinho, Vila Nova de Gaia, PRT

**Keywords:** endocarditis, fever, microbiology, stroke, thrombus

## Abstract

Infective endocarditis (IE) is a rare but serious life-threatening disease, often presenting with highly variable clinical symptoms. Risk factors for this condition include valvular heart disease, age, medical procedures, dental procedures, and intravenous drug use. Patients with IE may exhibit valve dysfunction, heart failure, or neurological complications such as stroke, the latter being caused by an embolus originating from valvular vegetations that occlude more distal vessels in the cerebral circulation. Diagnosis is based on the modified Duke criteria.

We report a case of a 20-year-old male patient who presented to the emergency department with the acute onset of aphasia, right-sided hemiparesis, and fever. Neurological examination revealed a National Institute of Health Stroke Scale score of 10, and computed tomography (CT) angiography confirmed a left middle cerebral artery occlusion. Since endocarditis was not known at the time of stroke diagnosis, the patient underwent thrombolysis. He then underwent thrombectomy with successful recanalization, and the retrieved thrombus was sent for microbiological analysis. Laboratory findings showed leukocytosis, elevated erythrocyte sedimentation rate, and elevated C-reactive protein, and both blood and thrombus cultures were positive for *Streptococcus anginosus*. An echocardiogram revealed a vegetation in the mitral valve, confirming the diagnosis of IE. The patient was treated with ceftriaxone, rifampicin, and gentamicin according to antibiotic susceptibility results, and underwent mitral valve replacement surgery, demonstrating a good clinical outcome with recovery from the neurological deficits.

This case highlights the importance of considering the diagnosis of IE in patients presenting with stroke and fever, as ischemic stroke can be an embolic complication of IE. It also emphasizes the potential role of microbiological analysis of clots retrieved through thrombectomy in identifying the infective agent, especially in patients where blood cultures are negative or inconclusive. Such identification might help select appropriate antibiotic therapy, although more studies are required to better define its role in patient care.

## Introduction

Infective endocarditis (IE) is a rare but serious infectious disease with high morbidity and mortality [1]. The incidence of IE ranges from three to fifteen cases per 100,000 persons per year [1,2]. This variation in incidence likely reflects differences in the prevalence of risk factors in the studied populations, such as structural heart disease, intravenous drug use, medical procedures, and comorbidities like diabetes or chronic kidney disease [3]. Clinical presentation is highly variable and depends on the underlying cardiac condition and preexisting comorbidities. Up to 90% of patients present with constitutional symptoms; however, other possible presentations include valve dysfunction, heart failure, or symptoms due to peripheral embolization, including stroke [4,5]. Stroke is reported to complicate up to 35% of IE cases, with the risk being higher for patients with large mobile vegetations and *Staphylococcus aureus* infection. However, other potential embolization sites include the bone, joints, kidney, and spleen [2]. Diagnosis of IE is based on the modified Duke criteria, which include clinical (fever, predisposing heart disease, and vascular phenomena), imaging (echocardiographic signs of vegetation or new-onset valvular insufficiency), immunological (rheumatoid factor, Osler's nodes, Roth's spots), and microbiological (blood cultures) criteria [2,4]. *S. aureus* is the most frequent cause of IE, representing approximately 26.6% of all cases, followed by viridans group streptococci (18.7%), other streptococci (17.5%), and enterococci (10.5%) [1]. In this case report, we address a case of large vessel occlusion stroke in a patient with endocarditis and explore the potential of microbiological analysis of the clot retrieved with thrombectomy in identifying the causative microorganism and guiding antibiotic therapy.

## Case presentation

A 20-year-old male patient with no significant medical history and no chronic medication presented to the emergency department with the sudden onset of aphasia and right-sided hemiparesis that started four hours earlier. The stroke fast-track pathway was activated on admission, and the patient was quickly evaluated. On physical examination, he had a respiratory rate of 25 breaths/minute, a blood pressure of 115/62 mmHg, a heart rate of 145 beats/minute, and a tympanic temperature of 40°C. On neurological examination, the patient was alert, with mild motor aphasia, right homonymous hemianopsia, right central facial paralysis, right-sided hemiparesis, and ipsilateral sensory loss. His National Institutes of Health Stroke Scale score was 10, compatible with a moderate stroke.

**Figure 1:**
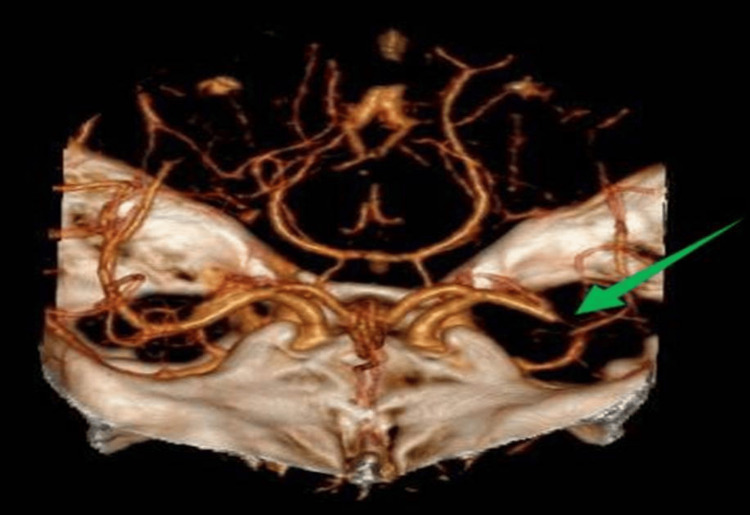
Occlusion of the M1 segment of the left middle cerebral artery, as shown by computed tomography angiography

Blood samples were sent for laboratory tests, and a computed tomography (CT) scan of the brain was performed, which showed only a small lateral lenticular infarct (Alberta Stroke Program Early CT Score of 9, consistent with mild ischemic changes and good prognosis). CT angiography demonstrated an occlusion of the distal M1 segment of the left middle cerebral artery, with no evidence of significant atherosclerotic plaques (Figure 1).

**Figure 2:**
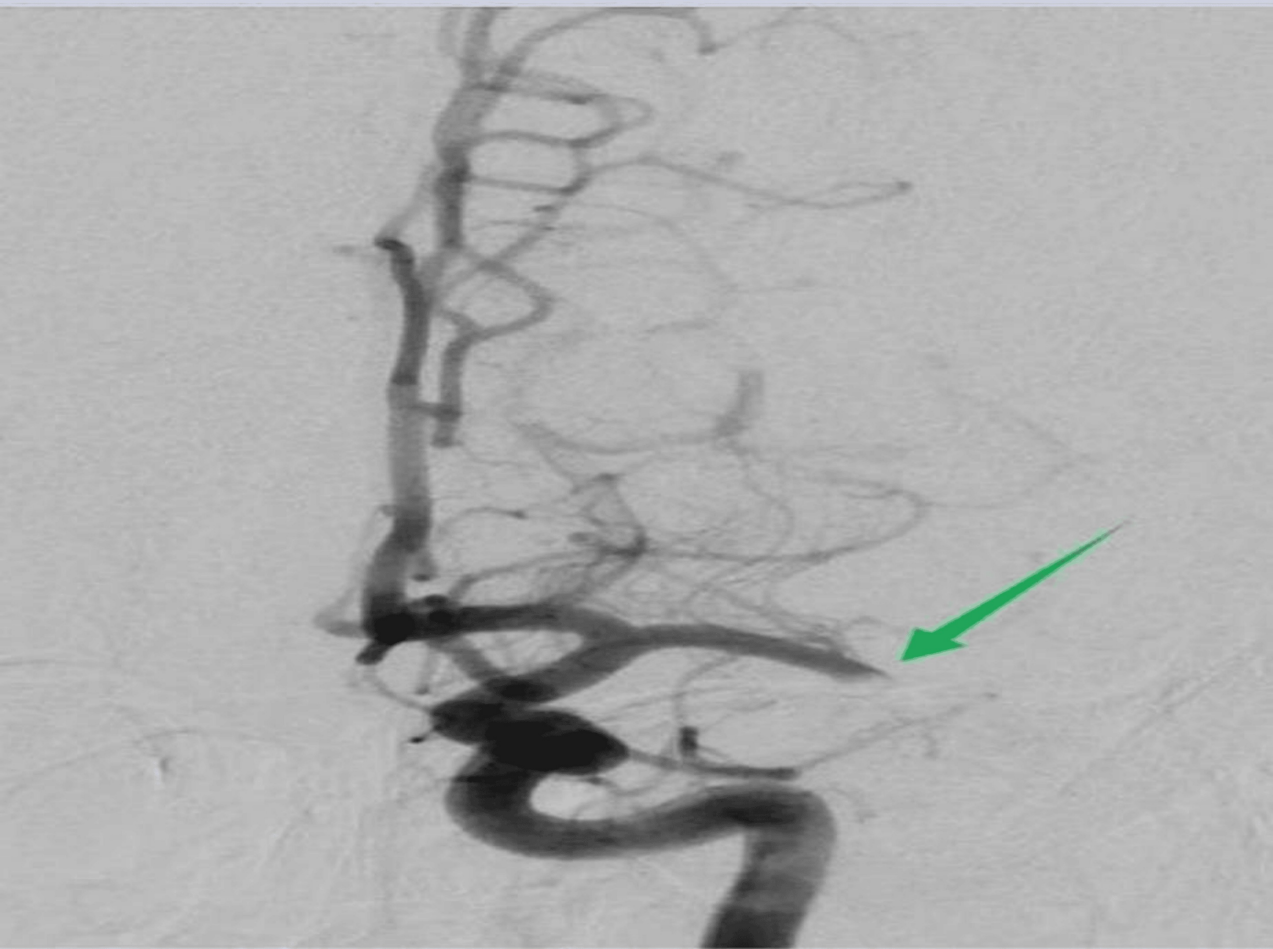
Angiography prior to thrombectomy

**Figure 3:**
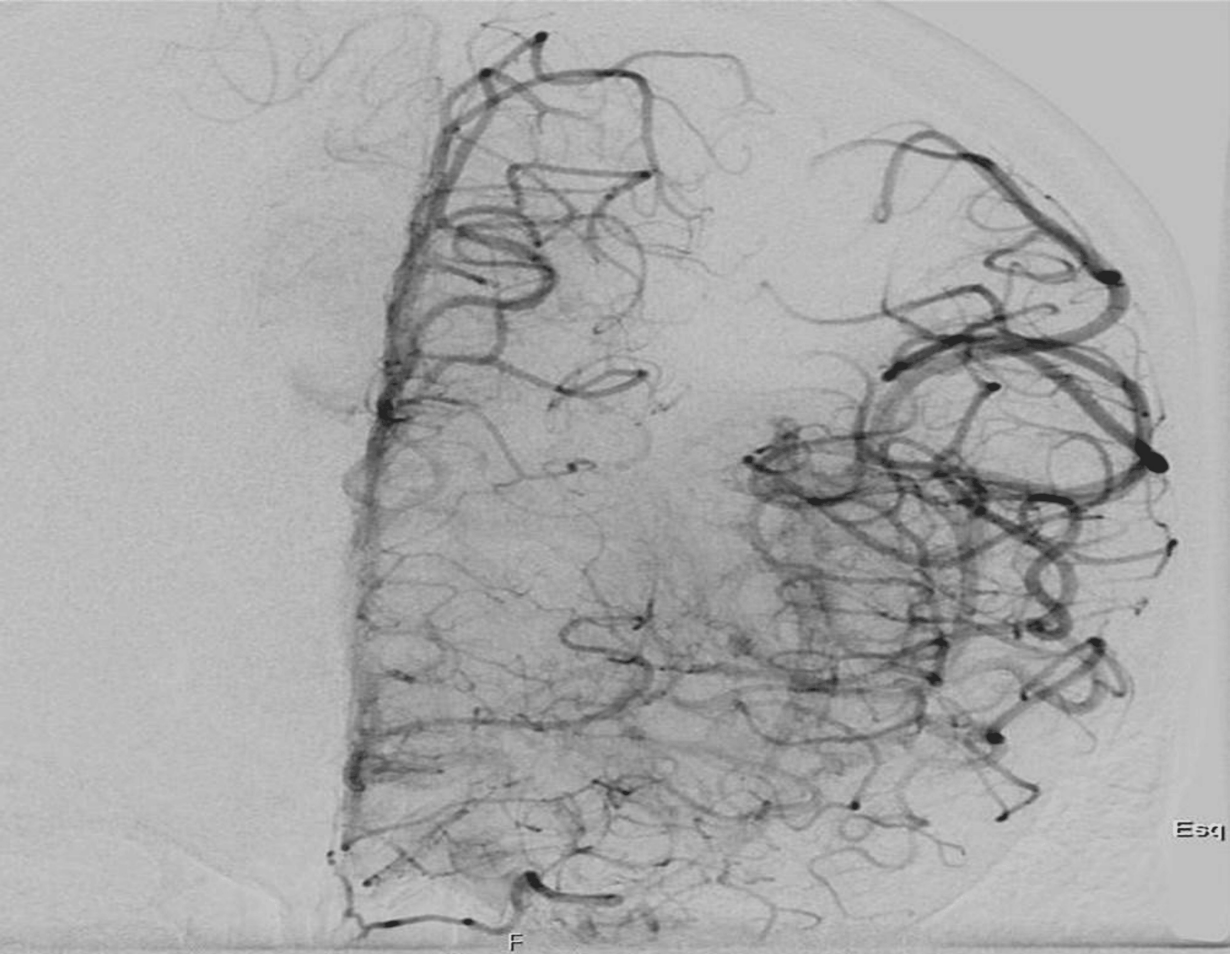
Angiography after thrombectomy with effective recanalization

Since the diagnostic criteria for IE were not met at this time, the patient underwent thrombolysis followed by thrombectomy with successful recanalization (Modified Treatment in Cerebral Infarction score of 3). The thrombus was sent for microbiological analysis due to clinical suspicion of endocarditis (Figures 2, 3). This diagnosis was suspected based on clinical criteria of fever and cerebral embolization, as laboratory tests and echocardiography results were unavailable.

**Figure 4:**
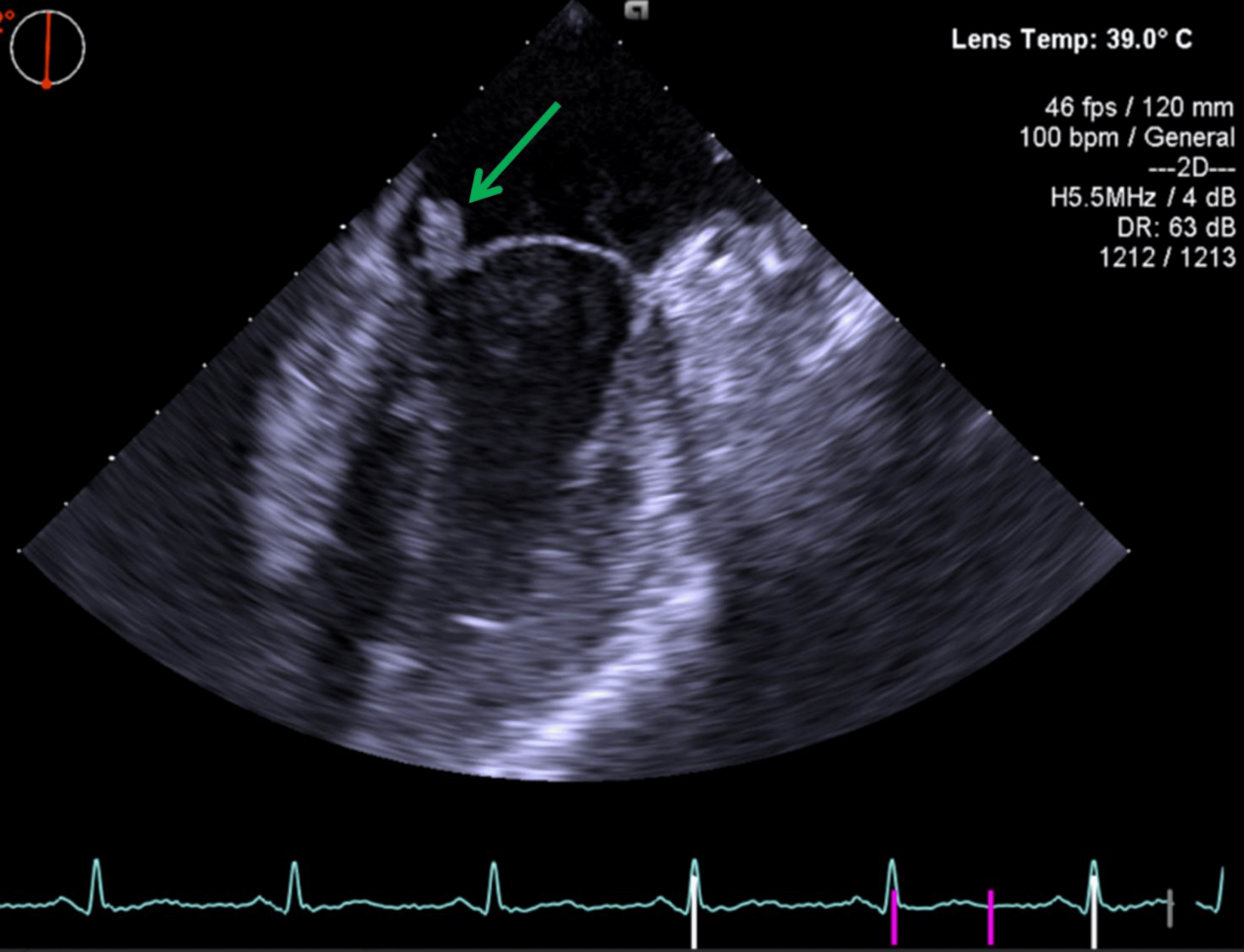
Vegetation on the leaflet of the mitral valve in transesophageal echocardiogram

Later on, the laboratory tests revealed a leukocytosis of 16,460/uL (3,600-11,000/uL) with neutrophilia, an elevated erythrocyte sedimentation rate of 64 mm/hour (0-20 mm/hour), and an elevated C-reactive protein of 14 mg/dL (0-0.5 mg/dL), with no other significant findings. Blood cultures were obtained before any antibiotics were administered, and a transthoracic echocardiogram was performed, which showed an echodense mass on the mitral valve leaflet causing mild-to-moderate mitral regurgitation. Empiric antibiotic therapy was started with vancomycin and gentamicin, and a transesophageal echocardiogram was requested, which confirmed the presence of an 11-mm vegetation on the mitral valve and better characterized the regurgitation as moderate to severe (Figure 4).

Blood cultures showed the presence of *Streptococcus anginosus* in all four bottles of the two sets of blood cultures taken, making the diagnosis of IE. The same microorganism was also isolated from the thrombus, and antibiotic therapy was adjusted to ceftriaxone, rifampicin, and gentamicin according to the antibiotic susceptibility testing. The patient underwent mitral valve replacement with a mechanical prosthesis on the fifteenth day of hospitalization, without complications. The timing of surgery was chosen in an attempt to strike a balance between preventing further embolization and minimizing the risk of hemorrhagic transformation of the cerebral infarct, given that anticoagulation was required for the procedure. Prior to surgery, dental care was optimized, as the patient's only identifiable risk factor for IE was poor oral health, with dental infections that had previously required tooth extraction. The patient demonstrated a good clinical evolution with complete recovery from the focal neurological deficits and completed six weeks of antibiotic therapy after blood cultures became negative.

Anticoagulation with warfarin was initiated during hospitalization due to the mechanical nature of the prosthetic valve, with enoxaparin bridging at the start of anticoagulation to further reduce the risk of stroke or systemic embolization. Regular monitoring of the international normalized ratio (INR) was used to ensure therapeutic anticoagulation levels and minimize the risk of bleeding, with a target INR of 2.5-3.5. In addition, continuous clinical and laboratory monitoring was performed, with serial complete blood counts, assessment of hemoglobin and hematocrit levels, and observation for any clinical signs of hemorrhage.

At one-year follow-up, the patient remained clinically stable, without any new neurological complications, and scored zero on the modified Rankin Scale. A follow-up echocardiogram demonstrated a normofunctioning mechanical prosthetic valve in the mitral position, preserved left ventricular systolic function, and no evidence of residual vegetation or other structural abnormalities.

## Discussion

It is estimated that 30% of patients with IE will develop CT-confirmed symptomatic cerebrovascular complications during their disease, but asymptomatic lesions are present in up to 80% [2]. Most clinically evident strokes are ischemic due to distal embolization of fragments from left-sided valvular vegetations, but cerebral hemorrhage can also occur due to the rupture of mycotic aneurysms [2,4,6,7]. Additionally, 10% of all strokes are associated with bacteremic infections, mostly endocarditis but also sepsis and meningitis. Although less common, strokes may be linked to viral or fungal infections [8,9].

An elevated body temperature in a patient presenting with acute stroke should raise concern about possible IE. If the diagnosis is strongly suspected, intravenous thrombolysis is not recommended due to the possible presence of mycotic aneurysms and inherent risk of cerebral hemorrhage, but mechanical thrombectomy can be beneficial in selected patients with large-vessel occlusion [2,10]. In this case report, the acute presentation of disease and the fact that diagnostic criteria were not gathered at the time of stroke treatment led to the administration of thrombolytic treatment, followed by thrombectomy. For patients with IE presenting with stroke, definitive blood culture results are typically not available at the time of disease presentation [2], and as such, clinicians should maintain a high level of suspicion for this diagnosis. Should the diagnosis have been more strongly considered on disease presentation, it would have been acceptable for this patient to proceed directly to thrombectomy and forego thrombolysis. This treatment, however, is also not exempt from risks such as cerebral hemorrhage, access site complications, distal embolization from clot dislodgment, vessel injury, and reperfusion injury [11].

Only approximately 40% of all blood cultures are positive in patients with endocarditis [12]. Potential causes for this low sensitivity include previous antibiotic treatment, IE caused by fastidious organisms, nonbacterial pathogens, or intracellular bacteria [13]. Although the diagnosis can still be made in patients with negative blood cultures, positive cultures can also assist in the selection of effective antimicrobial therapy. Our case report demonstrates that it is possible to isolate the culprit microorganism from clots obtained from thrombectomy, with other authors also reporting similar results [14,15]. Abdel-Wahed et al. reported a case of a 72-year-old woman with a bioprosthetic aortic valve who presented with a left middle cerebral artery stroke. This patient also presented with signs of infection, and endocarditis was diagnosed; the infectious agent was identified on clot analysis. Hernández-Fernández et al. [16] studied a consecutive sample of 65 patients who underwent thrombectomy for stroke over 24 months and identified bacteria in four, of these two being diagnosed with IE (50%), one with urinary tract infection, and one with respiratory septicemia. Another potentially useful test is histopathological analysis of the clot, which may allow visualization of infective bacteria with appropriate staining. This was elegantly demonstrated in a study by Bhaskar et al. involving four patients [17]. These results should be interpreted cautiously, given that they are drawn from case reports and small-scale observational studies involving a limited number of patients. Therefore, positive clot microbiology analysis should not be considered specific for IE but should be integrated with clinical, laboratory, and echocardiographic findings, which may also help determine whether the results reflect true infection or represent a contamination phenomenon. Such findings have led clinical societies to propose that embolic material retrieved from thrombectomy in such circumstances be sent for microbiological analysis [2], even though a positive microbiological growth in retrieved clots is not pathognomonic for IE.

To assist in diagnosing IE, the Duke criteria were proposed in 1994 and updated in 2000 (modified Duke Criteria). These criteria are divided into pathologic (histologic or microbiological analysis of valve tissue or abscess) and clinical (bacteremia, echocardiographic evidence of vegetations or new-onset valvular insufficiency, predisposing structural heart disease, fever, embolic, and immunological phenomena), the latter being further categorized into major and minor criteria. According to this classification, a definitive diagnosis of endocarditis can be made if patients have any pathologic criterion, two major clinical criteria, one major and three minor clinical criteria, or five minor clinical criteria [18,19]. However, these criteria were proposed at a time when thrombectomy was not performed for stroke treatment, as it is today. More recently, the modified Duke criteria have been updated by the International Society for Cardiovascular Infectious Diseases. Among other changes, this new diagnostic schema includes the isolation of an infectious microorganism from an arterial embolus as a pathologic criterion, while also emphasizing the need to carefully exclude bacterial contamination (particularly for commensal bacteria of the skin) [20]. Therefore, bacteriological analysis of the retrieved thrombus, in appropriately selected patients, can be useful in diagnosing endocarditis and can also guide antimicrobial therapy, especially in cases where blood cultures are negative. It should be noted, however, that no study to date has specifically evaluated the impact of this new criterion on the diagnosis of endocarditis in the context of stroke, nor can it be recommended as the standard of care for all stroke patients. Further studies are needed to clarify the role of microbiological analysis of thrombus in stroke thrombectomy, but it should be strongly considered in patients where IE is considered a potential underlying cause.

## Conclusions

Endocarditis should be suspected in patients presenting with stroke and fever, especially in those without cardiovascular risk factors who also exhibit a cardiac murmur. Thrombolysis is not recommended when endocarditis is strongly suspected. However, a definitive diagnosis is often not possible at the time of stroke presentation, as it requires correlation of clinical findings with echocardiogram imaging and blood cultures. The latter can even be negative, particularly in patients who recently received antibiotic therapy or are infected with fastidious microorganisms, intracellular pathogens, or nonbacterial pathogens. Thrombectomy remains an acceptable and effective treatment for patients with large vessel occlusion stroke, albeit it still carries a small risk of vessel injury or clot dislodgment. Microbiological analysis of the retrieved clot does not replace the need for blood cultures in the diagnosis of endocarditis but may aid in the identification of the infective agent and in guiding antibiotic therapy, particularly for patients with negative blood cultures.
